# Subclinical Respiratory Impairment and Quality of Life Among Non-Smoking Adults in Rural Chiang Mai, Thailand

**DOI:** 10.3390/jcm15031019

**Published:** 2026-01-27

**Authors:** Muhammad Samar, Tipsuda Pintakham, Muhammad Naeem Rashid, Nan Ei Moh Moh Kyi, Natthapol Kosashunhanan, Teetawat Santijitpakdee, Sawaeng Kawichai, Tippawan Prapamontol, Anurak Wongta

**Affiliations:** 1 School of Health Sciences Research, Research Institute for Health Sciences, Chiang Mai University, Chiang Mai 50200, Thailand; muhammad_samar@cmu.ac.th (M.S.); tipsuda_pintakham@cmu.ac.th (T.P.); muhammadnaeem_rashid@cmu.ac.th (M.N.R.); naneimohmoh_kyi@cmu.ac.th (N.E.M.M.K.); natthapol.ko@cmu.ac.th (N.K.); teetawat_santij@cmu.ac.th (T.S.); sawaeng.kaw@cmu.ac.th (S.K.); tippawan.prapamontol@cmu.ac.th (T.P.); 2 Research Institute for Health Sciences, Chiang Mai University, Chiang Mai 50200, Thailand; 3 Environmental, Occupational Health Sciences and NCD Research Group, Research Institute for Health Sciences, Chiang Mai University, Chiang Mai 50200, Thailand

**Keywords:** prevalence, subclinical respiratory impairment, spirometry, non-smokers, health-related quality of life, WHOQOL-BREF

## Abstract

**Background**: Subclinical respiratory impairment among non-smokers in regions with haze-affected regions is still under-recognized, particularly in low- and middle-income countries (LMICs). This study assessed the prevalence of subclinical respiratory impairment among non-smoking adults and examined its determinants and associations with health-related quality of life (HRQoL) in Chiang Mai, Thailand. **Methods**: In this cross-sectional study, 244 non-smoking adults (18–65 years) from three rural districts underwent standardized spirometry and completed the Thai WHOQOL-BREF-26. Subclinical impairment was defined as an FEV_1_/FVC < 0.70 or FVC < 80% predicted in the absence of symptoms. Demographic, occupational, and environmental information was obtained through structured questionnaires. Statistical analyses included non-parametric tests, univariate linear regression, and logistic regression. **Results**: A total of 37 participants (15.2%) had subclinical respiratory impairment. No demographic, occupational, or environmental factors such as sex, age, BMI category, agricultural work, marital status, and self-reported pollution exposure were found to be independently linked to impaired lung function. There was no correlation between spirometry indices and any WHOQOL-BREF domain. Elderly participants (>50 years) reported a higher level of physical and psychological HRQoL. Those with a higher Body Mass Index (BMI) were more likely to have a lower environmental quality of life. Farmers reported a better QoL, while women reported a lower QoL than men. **Conclusions**: Subclinical respiratory impairment occurs frequently in non-smoking rural adults exposed to haze pollution in Chiang Mai, and isn’t presently assessed by general HRQoL instruments. These findings support early spirometry screening for asymptomatic adults in polluted regions, as well as more stringent air cleanliness strategies to prevent the evolution towards overt respiratory pathology.

## 1. Introduction

Chronic obstructive pulmonary disease (COPD) ranks as the third most common cause of death globally, accounting for nearly 90% of deaths and more than 80% of the 315.5 million cases reported in low- and middle-income countries (LMICs) as of 2019, where the prevalence varies between 4.1% and 22.2% across different regions [1]. Chronic respiratory diseases are characterized as disorders affecting the airways and various components of the lungs [2], with prevalent conditions such as asthma, COPD, pulmonary fibrosis, infections of the respiratory tract, and lung cancer [3].

As a major contributor to illness and death, COPD generally leads to a gradual deterioration of health, frequent exacerbations, and ultimately respiratory failure. These factors contribute to a decline in quality of life (QoL), which is understood as a person’s overall assessment of their physical, mental, social, and environmental well-being [4]. This decline frequently results in higher rates of hospitalization and increased use of healthcare services [5].

Studies have shown a linear and statistically significant relationship between forced expiratory volume in one second (FEV_1_) and health-related quality of life (HRQoL) in patients with chronic respiratory diseases [6,7]. Additionally, ambient air pollution contributes to acute respiratory effects in populations of all ages [8]. This underscores the importance of examining subclinical respiratory impairment, which often remains undetected until irreversible damage has occurred.

Although lung function indices (e.g., FEV_1_) are associated with health status and quality of life at the population level, symptom burden and perceived health status are often only modestly related to spirometry in individual patients. Disease-specific instruments such as the St. George’s Respiratory Questionnaire (SGRQ) were developed to capture health status domains (symptoms, activity, and impacts) that are not fully explained by spirometry measures alone [9]. In clinical practice, COPD assessment and treatment decisions are guided by symptoms and exacerbation history in addition to airflow obstruction, and FEV_1_ is not used alone to guide pharmacological treatment choices [10].

Current literature demonstrates clear associations between air pollution exposure and respiratory health outcomes [8,11]. However, most existing studies have focused on symptomatic individuals or active smokers, leaving gaps in our understanding of respiratory risks among asymptomatic, non-smoking populations [12,13].

In Thailand, wildfires frequently occur in the northern, western, and upper northeastern areas [14], and have been linked to negative respiratory health effects [15]. Chiang Mai, located in the northern part of Thailand, has faced fluctuating elevated levels of air pollution since 2014. The province has been listed among the top five cities for respiratory illnesses like influenza and has sometimes been ranked within the top ten for cases of pneumonia. In 2019, it was reported to have a 24 h average PM_2.5_ concentration exceeding national standards [16], raising serious concerns about long-term respiratory health. Moreover, air pollution might contribute to increased rates of chronic lower respiratory disease in both hospital admissions and outpatient visits [17].

Despite these well-documented pollution levels, few studies have examined respiratory impairment across different occupational groups in this region [18,19]. Importantly, to date, no study has focused on subclinical respiratory impairment, defined as measurable reductions in lung function without clinical symptoms, among non-smoking populations in northern Thailand. This represents a critical knowledge gap, as early identification of respiratory impairment could allow timely intervention to prevent progression to chronic disease such as COPD.

While the impact of COPD on QoL has been extensively studied, the consequences of subclinical respiratory impairment (including clear reductions in exercise tolerance and impacts on daily functioning) remain poorly understood [20]. This study aims to address these gaps by estimating the prevalence of subclinical respiratory impairment among non-smokers in Chiang Mai, Thailand, and examining its relationship with quality of life using standardized spirometry and validated assessment tools.

## 2. Materials and Methods

### 2.1. Study Design and Participants

This cross-sectional research aimed to evaluate the prevalence of subclinical respiratory impairment among non-smokers in Chiang Mai, Thailand. It is an area characterized by frequent seasonal hazes with high ambient particulate matter concentrations due to biomass burning and air transboundary pollution. As such, background ambient air pollution exposure is very common across the study area.

A priori power analysis was performed using G*Power version 3.1.9.7 to determine the minimum sample size required for a linear multiple regression model (fixed model, R2 deviation from zero). The analysis indicated an effect size of f^2^ = 0.10, which aligns with a small-to-medium effect size as defined by Cohen’s criteria. The Type I error rate was established at α = 0.05, while the desired statistical power was set at 0.95, providing a high likelihood of identifying the anticipated effect. The model included seven predictors [21]. To increase the precision of estimates and to account for potential missing data, we aimed to recruit at least 243 participants.

Participants were recruited from the general community through community health hospitals. These hospitals facilitated community outreach work and initial health screening. Participants were not recruited from hospital outpatient or healthcare services.

All the participants were selected using convenience sampling by coordinating with the heads of local community health hospitals. To verify that participants were in a subclinical state, all individuals underwent a brief standardized health screening conducted by a trained healthcare professional. Subclinical status was defined by the absence of observable respiratory or cardiovascular symptoms, along with normal physiological parameters including resting oxygen saturation (SpO_2_), respiratory rate, heart rate, and breathing pattern. A structured medical history questionnaire was also used to confirm the absence of prior diagnoses, current respiratory complaints, or functional limitations. Participants who exhibited normal clinical parameters and reported no relevant symptoms were classified as subclinical and included in the study.

Inclusion criterion was healthy adults aged 18 to 65 years with no walking disabilities. Individuals were excluded if they were pregnant, had any congenital or acquired respiratory disease (such as asthma or chronic obstructive pulmonary disease), were active smokers, or were unable to perform spirometry testing. Individuals with known chronic respiratory disease, current respiratory symptoms, or regular use of respiratory medications were excluded. As participants were asymptomatic and community-dwelling adults, their daily activities were assumed to reflect normal functional status for their age group.

All participants underwent standardized assessments, including spirometry, to evaluate pulmonary function and exercise capacity. These procedures were carried out following established clinical guidelines to ensure consistency and reliability of data collection across all study sites.

### 2.2. Data Collection Procedures

Data collection involved a combination of standardized questionnaires, structured interviews, physical assessments, and functional testing. All procedures were conducted by a trained research team between 5 April and 5 May 2025, across three rural districts in Chiang Mai: Phrao, Chiang Dao, and Mae Na.

#### 2.2.1. Questionnaires and Interviews

Participants completed the WHOQOL-BREF-26, a validated Thai-language instrument for assessing quality of life [22]. Furthermore, a self-administered questionnaire with a structured format was utilized to gather information on demographic details, smoking habits, and medical history. Residents living near potential air pollution sources were assessed using self-reported information. Participants were asked whether their home or workplace was located near roads, agricultural areas, or forested land that could potentially generate air pollution. This variable was intended to reflect perceived proximity rather than quantitative or time-resolved exposure and therefore represents contextual exposure information rather than a direct measure of pollutant concentration. This information was verified and documented by trained personnel during face-to-face interviews.

#### 2.2.2. Assessment of HRQOL Scale

The WHOQOL-BREF questionnaire, which covers four domains, physical health, psychological state, social relationships, and environment, was used to measure the HRQOL scale through in-person interviews. The mean value of each domain’s items was used to calculate domain scores. Scores were then converted to a percentage system, ranging from 0 to 100, using the formula [23]:

Converted score = (original score − 4) × 100/16

Increased scores reflect an improved health-related quality of life.

#### 2.2.3. Physical and Functional Assessments

In accordance with standard protocols, measurements of body dimensions and blood flow were carried out in the morning. Prior to the measurements, participants removed their shoes and dressed in lightweight clothing. The calculation of body mass index (BMI) was performed by dividing weight in kilograms by height in meters squared (kg/m^2^) after both weight and height were recorded [24]. Brachial blood pressure (BP) and heart rate (HR) at rest were measured while the participants were seated, utilizing a validated oscillometric device (Omron Hem-8712, Omron Healthcare Co., Ltd., Kyoto, Japan) [25].

#### 2.2.4. Pulmonary Function Testing

Spirometry was conducted using a SpiroScout spirometer (Ganshorn Medizin Electronic GmbH, Niederlauer, Germany) in compliance with the American Thoracic Society (ATS) guidelines [26]. Under Royal Patronage, a qualified technician (Certificate No. 178/2566) from the Thoracic Society of Thailand performed all spirometry evaluations. The device underwent daily calibration in accordance with ATS standards.

Participants were asked to avoid large meals, exercise or smoke for 2 h before the test. For the test, the participants sat wearing a nose clip and were instructed to do Forced Vital Capacity (FVC) procedure following clear demonstration.

FVC, FEV_1_, and FEV1/FVC ratio were measured during the spirometry procedures. All the participants performed a minimum of three valid maneuvers, resulting in artifact-free curves with fast onset and continuous exhalation. The maximum FVC and FEV_1_ values were selected as long as the largest and second largest values differed by no more than 150 milliliters (mL).

Lung function impairment was defined as an FEV_1_/FVC ratio of less than 0.70 or an FVC% predicted value of less than 80% [26]. These spirometry results were then used to classify participants’ lung function as either normal or impaired.

### 2.3. Definition of Lung Function Impairment

Lung function was classified into two categories: normal and impaired. Impaired lung function was additionally defined as either obstructive (FEV_1_/FVC < 70%) or restrictive (FEV_1_/FVC ≥ 70% with FVC < 80% of the predicted value). These definitions adhered to global spirometry guidelines and considered individual factors like age, sex, and height.

Although the fixed ratio of FEV_1_/FVC < 70% is widely used, especially in large-scale or population-based studies, we acknowledge that this threshold may overestimate airflow limitation in older adults and underestimate it in younger individuals. Nevertheless, we adopted this criterion to maintain consistency with international practice and to enhance comparability with previous studies using similar methods.

In addition to the fixed spirometry cut-offs used in the primary analysis, sensitivity analyses based on Global Lung Function Initiative (GLI) lower limits of normal (LLN; z-score < −1.645) were conducted and are reported in the Supplementary Materials to assess the robustness of lung function impairment classification.

### 2.4. Statistical Analysis

Statistical evaluations were conducted with the use of IBM SPSS Statistics version 25. Continuous variables were represented as medians along with interquartile ranges (IQRs), whereas categorical variables were expressed in terms of frequencies and percentages. The Mann–Whitney U test was utilized to compare continuous variables, while the Chi-square test was used for categorical data. A *p*-value of less than 0.05 was considered statistically significant.

Associations between participant characteristics (sex, age group, BMI category, occupation, marital status, and self-reported proximity to pollution sources) and spirometry indices (FEV_1_% predicted, FVC% predicted, and FEV_1_/FVC ratio) or WHOQOL-BREF domain scores (physical, psychological, social, and environmental) were explored using non-parametric tests and univariable linear regression. Variables with *p* < 0.10 in univariable analyses were subsequently included in multivariable models to assess independent associations. Results are presented as unstandardized β coefficients with 95% confidence intervals (CIs). As a sensitivity analysis, Spearman’s rank correlation was performed to assess the robustness of associations between spirometry parameters and WHOQOL-BREF domain scores.

Binary logistic regression was used to identify factors associated with subclinical respiratory impairment, defined as FEV_1_/FVC < 0.70 and/or FVC < 80% predicted, in the absence of symptoms. The following covariates included in the model were based on prior literature and clinical relevance: sex, age category (≤50 vs. >50 years), BMI category, marital status, agricultural occupation, and proximity to pollution sources.

### 2.5. Ethical Approval and Consent

All individuals involved in the study signed a consent form before the data was gathered. Ethical approval was granted by the Research Institute for Health Sciences (RIHES) at Chiang Mai University (Approval No. 9/67), covering the period from 16 May 2024.

Confidentiality was maintained by using coded identifiers, secure data storage, and anonymizing data before analysis. All research personnel received training on privacy protocols to protect participant information during the administration of spirometry and questionnaires.

Any issues that arose during data collection, like nervousness regarding spirometry, were managed through pre-test counseling and a clear explanation of the procedures during the consent process.

## 3. Results

### 3.1. Prevalence of Subclinical Respiratory Impairment and Participant Characteristics

Among 244 participants, 37 (15.2%) had reduced lung function, while 207 (84.8%) had normal spirometry results.

Baseline characteristics by lung function status are shown in Table 1. The median age was 53 years (IQR: 45–59), with no significant difference between those with normal lung function and those with lung impairment (*p* = 0.687). Most participants were female (79.8%). The distribution of sex did not differ significantly between participants with normal lung function and those with lung impairment (female: 78.3% vs. 75.7%; *p* = 0.727). No significant differences were observed between groups with respect to body mass index (BMI) (*p* = 0.650), agricultural occupation (*p* = 0.673), or self-reported living near potential air pollution sources (*p* = 0.215).

Health-related quality of life (WHOQOL-BREF) domain scores did not differ significantly across groups in the physical (*p* = 0.532), psychological (*p* = 0.436), social (*p* = 0.745), or environmental (*p* = 0.326) domains. In summary, demographic factors that were not significantly associated with subclinical respiratory impairment in this population.

Comparable findings were observed when lung function impairment was defined using LLN criteria, as shown in Supplementary Table S1.

### 3.2. Associations of Participant Characteristics with Quality of Life Scores

Associations between participant characteristics and WHOQOL-BREF scores are presented in Table 2.

In terms of quality-of-life scores, older adults (>50 years) reported better physical and psychological health (both *p* < 0.01). Farmers also reported higher environmental scores than non-farmers (*p* < 0.01). Body mass index (BMI) category was not associated with physical, psychological, or social domain scores (all *p* > 0.05). However, a significant difference was observed in the environmental domain, with BMI categories differing in environmental quality-of-life scores (*p* = 0.033). Marital status was significantly associated with the social domain of WHOQOL-BREF (*p* = 0.037), whereas no significant differences were observed in the physical (*p* = 0.291), psychological (*p* = 0.356), or environmental (*p* = 0.258) domains.

No statistically significant differences in WHOQOL-BREF domain scores were observed according to self-reported Living near potential air pollution sources across all domains (all *p* > 0.05).

Overall, age, marital status, BMI category, and occupation showed modest associations with selected quality-of-life domains, whereas sex and perceived proximity to air pollution sources were not associated with WHOQOL-BREF scores in this population.

Analyses of spirometry parameters across participant characteristics showed no statistically significant differences and are presented in Supplementary Table S2.

### 3.3. Factors Associated with Lung Function Impairment

In univariable analyses, no demographic, occupational, or environmental factors were significantly associated with subclinical lung function impairment.

Similarly, in multivariable logistic regression models adjusting for age, sex, BMI, farming status, and living near potential air pollution sources, no factors were significantly associated with lung function impairment (Table 3).

Sensitivity analyses using LLN-based definitions of lung function impairment yielded consistent findings, with no variables showing statistically significant associations (Table S3).

### 3.4. Associations Between Demographic, Occupational, and Environmental Factors and WHOQOL-BREF Domains

Table 4 presents the results of univariate linear regression analyses examining associations between participant characteristics and WHOQOL-BREF domain scores.

In univariate analyses, older age (>50 years) was positively associated with quality-of-life scores in the physical domain (β = 2.91, 95% CI: 0.84–4.98) and psychological domain (β = 4.69, 95% CI: 1.94–7.44). The association with the social relationship domain did not reach statistical significance (β = 3.75, 95% CI: −0.10 to 7.60).

Body mass index (BMI) category was not associated with physical, psychological, or social domain scores; however, obesity was negatively associated with the environmental domain (β = −2.96, 95% CI: −4.80 to −1.12). Marital status was associated with the social relationship domain, with widowed participants reporting lower social scores compared to married participants (β = −3.69, 95% CI: −7.13 to −0.25).

Agricultural occupation was positively associated with psychological (β = 2.81, 95% CI: 0.01–5.60), social relationship (β = 5.35, 95% CI: 1.53–9.17), and environmental domains (β = 3.63, 95% CI: 0.50–6.76). No significant associations were observed for sex or residential proximity to air pollution sources across WHOQOL-BREF domains in univariate models.

Multivariable linear regression analyses were subsequently performed to adjust for potential confounding factors. As shown in Supplementary Table S4, several domain-specific associations identified in univariate analyses remained statistically significant after adjustment, although effect estimates were generally attenuated across domains.

Overall, these findings indicate that selected demographic and occupational characteristics were associated with specific WHOQOL-BREF domains, with more conservative associations observed after multivariable adjustment.

### 3.5. Relationship Between Lung Function and Quality of Life Domains

The relationship between spirometry parameters and WHOQOL-BREF domain scores was examined using univariate linear regression (Table 5). No statistically significant associations were found between FEV_1_% predicted, FVC% predicted, or FEV_1_/FVC ratio and any of the quality-of-life domains.

Specifically, none of the spirometry indices were associated with physical health (FEV_1_% predicted: β = −0.03, *p* = 0.609; FVC% predicted: β = −0.04, *p* = 0.698; FEV_1_/FVC: β = 0.00, *p* = 0.609). Similarly, no significant associations were observed for the psychological, social relationship, or environmental domains (all *p* > 0.05).

Additional correlation analyses yielded consistent results, showing no meaningful associations between spirometry parameters and WHOQOL-BREF domain scores (Supplementary Table S5).

Overall, spirometry measures were not associated with WHOQOL-BREF domain scores in this population.

## 4. Discussion

This study examined the prevalence and determinants of subclinical respiratory impairment among non-smoking adults residing in rural districts of Chiang Mai, Thailand, and explored potential associations between lung function and HRQoL. The key findings were as follows: the prevalence of subclinical respiratory impairment was 15.2%; no demographic, occupational, or environmental factors were independently associated with impaired lung function; and factors including age, farming status, BMI, and perceived pollution exposure were not significantly associated with lung impairment. Additionally, no significant relationship was found between spirometry results and WHOQOL-BREF domain scores. The lack of relationship between the spirometry parameters and the domains of quality of life remained consistent after the linear regression and non-parametric correlation tests.

### 4.1. Prevalence of Subclinical Respiratory Impairment

The prevalence of subclinical impairment in this non-smoking population (15.2%) is consistent with reports from other Asian populations, where 10–20% of adults exhibit reduced lung function despite having no respiratory symptoms or smoking history [27,28]. This early-stage impairment is often undetected in routine health surveillance, particularly in low-resource settings where diagnostic testing is limited.

Chiang Mai is a region severely affected by recurrent seasonal haze and PM_2.5_ pollution, largely driven by agricultural burning and transboundary smoke [29,30]. Studies show that even short-term increases in PM_2.5_, NO_2_, and ozone, even at concentrations below international standards, can be associated with measurable reductions in FEV_1_ and FVC among non-smokers [31,32]. It should be noted that, by definition, a proportion of individuals without respiratory disease will have spirometry values below fixed cut-offs, and the estimated prevalence of impairment depends on the reference equations and thresholds applied. In the present study, sensitivity analyses using GLI-based LLN yielded similar patterns, supporting that the observed prevalence likely reflects early functional variation rather than overt respiratory disease. Given this environmental context, the presence of subclinical impairment in our study population is biologically plausible. Chronic exposure to particulate matter may contribute to airway inflammation, oxidative stress, and small airway dysfunction years before symptoms develop [33,34,35].

### 4.2. Determinants of Lung Function Impairment

Although findings of previous studies have suggested that women might be more vulnerable to the respiratory effects of air pollution and biomass exposure [36], no significant associations between sex and subclinical lung function impairment were observed in the present study in either univariable or multivariable logistic regression analyses. Proposed biological and anatomic mechanisms underlying sex-related susceptibility, such as narrower airways, increased deposition of particles per unit lung volume, and hormonal modulation of airway inflammation, have been described in the literature [37,38,39]. In rural Thai settings, women may also experience higher cumulative exposure due to greater time spent near combustion sources such as cooking or field burning [40].

Importantly, indoor air pollution exposure was not directly measured in this study, and all participants resided in haze-affected rural areas characterized by elevated background ambient air pollution. The exposure classification therefore reflected perceived proximity to potential local sources rather than a contrast between polluted and non-polluted environments. The absence of significant associations in the present analysis suggests that sex-related differences may not be consistently detectable in community-based, asymptomatic populations, particularly in settings where exposure contrast is limited.

In our study, however, the absence of independent associations between lung function impairment and demographic or occupational factors suggests that observed variations should be interpreted cautiously. Part of the observed difference may reflect anatomical or behavioral factors that were not fully captured in our dataset, rather than a direct biological susceptibility. Notably, most individuals with reduced lung function in this study were asymptomatic, indicating that such impairment may remain clinically silent unless actively screened. This underscores the relevance of detecting subclinical respiratory dysfunction in high-risk, non-smoking populations exposed to environmental pollutants, where early intervention may be feasible even before symptoms emerge.

Other factors—age, BMI, agricultural occupation, and proximity to pollution sources—did not show significant associations with lung impairment. The lack of association with age contrasts with existing evidence of age-related lung function decline due to physiological changes [41]. However, this may be due to the relatively narrow age range (18–65 years) and the exclusion of individuals with clinically diagnosed respiratory disease, which may have limited the detection of age-related patterns [42,43]. A similar caution applies to the inverse trend seen with BMI, which may reflect residual confounding or the influence of body composition not captured in this dataset.

During the biomass-burning season, PM_2.5_ levels are elevated across wide areas of northern Thailand [44]. This widespread exposure may explain the lack of significant differences by occupation or residence near local pollution sources. Self-reported exposure may also be an imprecise measure, as individuals’ perceptions often do not reflect actual pollution levels [45]. Future studies could benefit from objective exposure assessment using personal PM_2.5_ monitors or spatially resolved air quality data [46,47].

Although obese participants and farmers showed slightly higher FEV_1_/FVC ratios in continuous analyses, these differences did not translate into an increased prevalence of lung function impairment. This may be partly explained by the “obesity paradox,” whereby moderate obesity can improve FVC and mask impairment [48]. Similarly, farmers’ higher FEV_1_/FVC ratios may reflect a “healthy worker effect,” wherein individuals with better lung function remain employed in physically demanding jobs. Previous studies have shown lower lung function among former farmers compared to those still actively employed [49].

### 4.3. Relationship Between Lung Function and Quality of Life

Contrary to the study hypothesis, spirometry indices were not significantly associated with WHOQOL-BREF scores. This finding indicates a lack of observable association between lung function measures and perceived quality of life in this population, which is expected among asymptomatic individuals. Previous research indicates that quality-of-life deterioration becomes more apparent once symptoms such as dyspnea, cough, or reduced exercise tolerance emerge, or when obstruction progresses to moderate or severe stages [50].

It is also possible that the WHOQOL-BREF, as a general QoL instrument, lacks sensitivity to detect early impairments. Disease-specific tools such as the St. George’s Respiratory Questionnaire (SGRQ) or the COPD Assessment Test (CAT) may better capture subtle changes in early disease but were not utilized in this study [51].

Interestingly, some demographic and lifestyle factors were associated with QoL. Participants aged > 50 years reported better physical and psychological QoL, which may reflect stable health status in early older adulthood [52]. Conversely, higher BMI was linked to lower physical and environmental QoL, consistent with established associations between obesity and reduced physical functioning [53]. Farmers reported higher environmental QoL, possibly due to greater satisfaction with rural living or perceived connectedness to natural surroundings [54]. In contrast, variations in environmental quality-of-life scores should be interpreted cautiously, as these patterns were not consistently observed after multivariable adjustment and may reflect contextual or psychosocial factors rather than disease-specific effects [55,56].

### 4.4. Public Health and Clinical Implications

The detection of subclinical respiratory impairment among non-smokers highlights the importance of early screening, especially in regions affected by air pollution. As these impairments were not reflected in QoL scores, relying solely on symptoms or perceived health status could delay diagnosis until disease progression occurs.

Community-based screening programs targeting asymptomatic adults in pollution-prone areas may help identify at-risk individuals before symptoms appear. Although no demographic or occupational factors were independently associated with impairment in multivariable analyses, the findings reinforce the need for strengthened air quality management in northern Thailand. Interventions such as reducing biomass burning, implementing early-warning systems, improving indoor ventilation, and promoting protective behaviors (e.g., mask use) could help prevent the transition from subclinical to clinically significant disease [57,58].

These findings underscore the importance of early screening in asymptomatic adults living in pollution-affected regions, where subclinical impairment may remain undetected without objective lung function testing.

### 4.5. Strengths and Limitations

This study has several strengths, including the use of standardized spirometry (ATS/ERS guidelines), validated QoL instruments, and a focus on non-smoking rural adults, a group often underrepresented in air pollution research. However, limitations must be acknowledged. Firstly, the cross-sectional design limits causal inference. Secondly, pollution exposure was assessed via self-report rather than direct measurement. Thirdly, the sample was predominantly female, which may limit generalizability. Fourthly, the WHOQOL-BREF may not be sensitive to early respiratory-related changes in QoL. Lastly, the use of fixed cut-offs to define impairment could misclassify individuals near threshold values; however, sensitivity analyses applying LLN criteria yielded consistent findings, supporting the robustness of the observed prevalence estimates and associations [59].

## 5. Conclusions

In conclusion, this study identified a 15.2% prevalence of subclinical respiratory impairment among non-smoking adults in rural Chiang Mai. No demographic, occupational, or contextual environmental factors were significantly associated with impaired lung function in the multivariable analysis. Despite measurable reductions in spirometry indices, no significant associations were observed with quality-of-life domains, suggesting that early-stage impairment may remain both clinically and subjectively silent.

Although this study did not demonstrate a direct association between lung function impairment and the air pollution indicator used, the presence of subclinical impairment in a haze-affected population underscores the importance of proactive respiratory health surveillance and preventive public health measures. Continued efforts to improve air quality control and health education may help protect respiratory health in regions with chronically elevated ambient pollution. Future research incorporating longitudinal designs and objective exposure assessments is needed to clarify the trajectory from subclinical impairment to symptomatic disease.

## Figures and Tables

**Table 1:** Demographic and Health Characteristics of Participants by Lung Function Category.

Variable	Total	Normal Lung Function	Lung Impairment	*p*-Value
	(n = 244)	(n = 207)	(n = 37)	
Age (years), median (IQR)	53 (45.25–59)	53 (46–59)	53 (43–60)	0.687
Sex				
Female, n (%)	190 (77.9)	162 (78.3)	28 (75.7)	0.727
Male, n (%)	54 (22.1)	45 (21.7)	9 (24.3)	
BMI (kg/m^2^), median (IQR)	25.3 (23.025–28.325)	25.2 (23–28)	25.8 (22.95–29.85)	0.650
Farmers				0.673
Yes, n (%)	144 (59.0)	121 (58.5)	23 (62.2)	
No, n (%)	100 (41.0)	86 (41.5)	14 (37.8)	
Living near potential air pollution sources				0.215
Yes, n (%)	154 (63.1)	134 (64.7)	20 (54.1)	
No, n (%)	90 (36.9)	73 (35.3)	17 (45.9)	
WHOQOL-BREF Scores.				
Physical health	57.14 (53.57–64.29)	57.14 (53.57–64.29)	60.71 (53.57–64.29)	0.532
Psychological health	58.33 (54.17–66.67)	58.33 (54.17–66.67)	58.33 (50.00–66.67)	0.436
Social health	58.33 (50.00–75.00)	58.33 (50.00–75.00)	58.33 (50.00–75.00)	0.745
Environmental health	62.50 (56.25–71.88)	62.50 (56.25–68.75)	65.63 (56.25–75.00)	0.326

Abbreviations: IQR, interquartile range; BMI, body mass index. ***p***-values were calculated using the Mann–Whitney U test for continuous variables and the Chi-square test for categorical variables. Statistical significance was considered at ***p*** < 0.05.

**Table 2:** Associations of Participant Characteristics with WHOQOL-BREF Scores.

Variable	N	BREFF–26 Domain Score Median (IQR)			
		Physical	Psychological	Social	Environmental
Gender					
Male	54	57.1 (52.7–60.7)	62.5 (54.2–70.8)	66.7 (56.3–75.0)	65.6 (59.4–75.0)
Female	190	60.7 (53.6–64.3)	58.3 (50.0–66.7)	58.3 (50.0–75.0)	62.5 (56.3–68.8)
*p*-value		0.204	0.107	0.220	0.056
Age (Years)					
≤50	101	57.1 (50.0–60.7)	58.3 (50.0–66.7)	58.3 (50.0–75.0)	62.5 (54.7–71.9)
>50	143	60.7 (53.6–64.3)	62.5 (54.2–66.7)	66.7 (50.0–75.0)	65.6 (56.3–68.8)
*p*-value		0.005 **	0.001 **	0.087	0.882
BMI(kg/m^2^)					
Normal	58	60.7 (53.6–64.3)	62.5 (54.2–70.8)	66.7 (50.0–75.0)	65.6 (59.4–75.0)
Overweight	53	60.7 (53.6–60.7)	58.3 (52.1–66.7)	58.3 (50.0–75.0)	62.5 (59.4–71.9)
Obese	133	57.1 (50.0–64.3)	58.3 (52.1–66.7)	58.3 (50.0–75.0)	62.5 (53.1–68.8)
*p*-value		0.219	0.319	0.435	0.033 *
Marital status					
Single	41	60.7 (53.6–64.3)	62.5 (54.2–66.7)	66.7 (58.3–75.0)	65.6 (59.4–73.4)
Married	170	57.1 (52.7–64.3)	58.3 (54.2–66.7)	58.3 (50.0–75.0)	62.5 (56.3–69.5)
Widowed/divorced	33	60.7 (55.4–64.3)	58.3 (50.0–70.8)	58.3 (50.0–75.0)	65.6 (56.3–68.8)
*p*-value		0.291	0.356	0.037 *	0.258
Occupation					
Farmer	144	57.1 (53.6–63.4)	58.3 (50.0–66.7)	58.3 (50.0–75.0)	62.5 (53.1–68.8)
Non-Farmer	100	60.7 (53.6–64.3)	62.5 (54.2–66.7)	66.7 (58.3–75.0)	65.6 (59.4–71.9)
*p*-value		0.062	0.051	0.007 **	0.02 *
Living near potential air pollution sources					
No	90	60.7 (53.6–64.3)	62.5 (54.2–66.7)	66.7 (50.0–75.0)	62.5 (56.3–68.8)
Yes	154	57.1 (53.6–60.7)	58.3 (53.1–66.7)	58.3 (50.0–75.0)	65.6 (56.3–71.9)
*p*-value		0.145	0.079	0.376	0.231

Abbreviations: BMI, Body mass index. Mann–Whitney U test and Kruskal–Wallis test were used to check the significance. Statistical significance was considered at * *p* < 0.05 and ** *p* < 0.01.

**Table 3:** Multivariable logistic regression analysis of factors associated with subclinical lung function impairment.

Variable	Odds Ratio (OR)	95% CI (Lower–Upper)	*p*-Value
Gender (male)	1.292	0.533, 3.132	0.570
Age > 50 (vs. ≤50)	1.067	0.502, 2.267	0.867
BMI category	1.026	0.666, 1.581	0.907
Marital status	1.315	0.669, 2.582	0.427
Farmer (vs. non-farmer)	0.934	0.439, 1.986	0.860
Living near potential air pollution sources	0.62	0.300, 1.280	0.196

Abbreviations: OR, odds ratio; CI, confidence interval; *p* < 0.05, by logistic regression analysis. Adjusted for age, sex, BMI, farming status, and proximity to potential air pollution sources.

**Table 4:** Univariate Linear Regression Between Participant Characteristics and WHOQOL-BREF Domain Scores.

Variable	Physical Health	Psychological	Social Relationships	Environmental
	β (95% CI)	β (95% CI)	β (95% CI)	β (95% CI)
Gender				
1 = male	−1.46 (−3.95, 1.03)	2.08 (−1.25, 5.41) ^¶^	1.94 (−2.65, 6.53)	3.17 (−0.56, 6.91)
Age > 50 years				
1 = Yes	2.91 (0.84, 4.98) * ^¶^	4.69 (1.94, 7.44) * ^¶^	3.75 (−0.10, 7.60)	0.35 (−2.81, 3.52)
BMI				
1 = Overweight				
2 = Obese	−1.22 (−2.46, 0.02)	−1.33 (−2.99, 0.34)	−1.82 (−4.10, 0.48)	−2.96 (−4.80, −1.12) * ^¶^
Marital Status				
1 = Married				
2 = Widowed	−0.03 (−1.91, 1.85)	−0.80 (−3.32, 1.72)	−3.69 (−7.13, −0.25) * ^¶^	−2.12 (−4.94, 0.71)
Agricultural Worker				
1 = Yes	1.82 (−0.27, 3.91)	2.81 (0.01, 5.60) * ^¶^	5.35 (1.53, 9.17) * ^¶^	3.63 (0.50, 6.76) * ^¶^
Living near potential air pollution sources				
1 = Yes	−1.58 (−3.71, 0.56)	−2.81 (−5.66, 0.04)	−1.37 (−5.32, 2.59)	0.51 (−2.73, 3.74)

Abbreviations: CI, confidence interval; BMI, body mass index. *p*-values were calculated using univariate linear regression. Ref = 0 indicates the reference category for dummy-coded variables. Statistical significance was considered at * *p* < 0.05 and ^¶^ indicates variables that remained significant in multivariable linear regression.

**Table 5:** Univariate Linear Regression Between Spirometry Parameters and WHOQOL-BREF Domain Scores.

Outcome Domain	FEV1% Predicted	*p*-Value	FVC% Predicted	*p*-Value	FEV1/FVC	*p*-Value
	β Coefficient (95% CI)		β Coefficient (95% CI)		β Coefficient (95% CI)	
Physical health	−0.03 (−0.10, 0.04)	0.609	−0.04 (−0.27, 0.18)	0.698	0.00 (−0.00, 0.00)	0.609
Psychological health	−0.01 (−0.17, 0.16)	0.957	−0.03 (−0.19, 0.14)	0.75	0.00 (−0.00, 0.00)	0.267
Social relationships	−0.02 (−0.14, 0.10)	0.743	0.01 (−0.11, 0.13)	0.833	−0.00 (−0.00, 0.00)	0.053
Environmental domain	−0.04 (−0.19, 0.11)	0.598	−0.03 (−0.18, 0.12)	0.722	0.00 (−0.00, 0.00)	0.207

Abbreviations: β (Beta): Unstandardized regression coefficient, FEV_1_: Forced expiratory volume in one second, FVC: Forced vital capacity, FEV1/FVC: ratio.

## Data Availability

The original contributions presented in the study are included in the article; further inquiries can be directed at the corresponding author.

## Supplementary Materials

The following supporting information can be downloaded at: https://www.mdpi.com/article/10.3390/jcm15031019/s1, Table S1. Demographic and Health Characteristics of Participants by Lung Function Category. Table S2. Associations of Participant Characteristics with Spirometry and WHOQOL-BREF Scores. Table S3. Multiple Logistic Regression Analysis of Factors Associated with Lung Function Impairment. Table S4. Linear Regression Between Participant Characteristics and WHOQOL-BREF Domain Scores. Table S5. Spearman’s rank correlation between spirometry parameters and WHOQOL-BREF domain scores (N = 244).
